# Bicarbonate-Stimulated Membrane Reorganization in Stallion Spermatozoa

**DOI:** 10.3389/fcell.2021.772254

**Published:** 2021-11-17

**Authors:** Paula Piccolo Maitan, Elizabeth G. Bromfield, Romy Hoogendijk, Miguel Ricardo Leung, Tzviya Zeev-Ben-Mordehai, Chris H. van de Lest, Jeroen W. A. Jansen, Bart Leemans, José Domingos Guimarães, Tom A. E. Stout, Bart M. Gadella, Heiko Henning

**Affiliations:** ^1^ Department of Clinical Sciences, Faculty of Veterinary Medicine, Utrecht University, Utrecht, Netherlands; ^2^ Department of Veterinary Medicine, Universidade Federal de Viçosa, Viçosa, Brazil; ^3^ Department of Biomolecular Health Science, Faculty of Veterinary Medicine, Utrecht University, Utrecht, Netherlands; ^4^ Priority Research Centre for Reproductive Science, The University of Newcastle, Callaghan, NSW, Australia; ^5^ Cryo-Electron Microscopy, Bijvoet Centre for Biomolecular Research, Utrecht University, Utrecht, Netherlands; ^6^ Department of Population Health Science, Faculty of Veterinary Medicine, Utrecht University, Utrecht, Netherlands

**Keywords:** spermatozoa, capacitation, membrane, lipid, bicarbonate (HCO_3_−), fertilization, equine, adenylyl cyclase

## Abstract

Classical *in vitro* fertilization (IVF) is still poorly successful in horses. This lack of success is thought to be due primarily to inadequate capacitation of stallion spermatozoa under *in vitro* conditions. In species in which IVF is successful, bicarbonate, calcium, and albumin are considered the key components that enable a gradual reorganization of the sperm plasma membrane that allows the spermatozoa to undergo an acrosome reaction and fertilize the oocyte. The aim of this work was to comprehensively examine contributors to stallion sperm capacitation by investigating bicarbonate-induced membrane remodelling steps, and elucidating the contribution of cAMP signalling to these events. In the presence of capacitating media containing bicarbonate, a significant increase in plasma membrane fluidity was readily detected using merocyanine 540 staining in the majority of viable spermatozoa within 15 min of bicarbonate exposure. Specific inhibition of soluble adenylyl cyclase (sAC) in the presence of bicarbonate by LRE1 significantly reduced the number of viable sperm with high membrane fluidity. This suggests a vital role for sAC-mediated cAMP production in the regulation of membrane fluidity. Cryo-electron tomography of viable cells with high membrane fluidity revealed a range of membrane remodelling intermediates, including destabilized membranes and zones with close apposition of the plasma membrane and the outer acrosomal membrane. However, lipidomic analysis of equivalent viable spermatozoa with high membrane fluidity demonstrated that this phenomenon was neither accompanied by a gross change in the phospholipid composition of stallion sperm membranes nor detectable sterol efflux (*p* > 0.05). After an early increase in membrane fluidity, a significant and cAMP-dependent increase in viable sperm with phosphatidylserine (PS), but not phosphatidylethanolamine (PE) exposure was noted. While the events observed partly resemble findings from the *in vitro* capacitation of sperm from other mammalian species, the lack of cholesterol removal appears to be an equine-specific phenomenon. This research will assist in the development of a defined medium for the capacitation of stallion sperm and will facilitate progress toward a functional IVF protocol for horse gametes.

## Introduction

To fertilize an oocyte, spermatozoa must undergo a process called capacitation, which begins when the spermatozoa enter the female reproductive tract, or are exposed to specific *in vitro* capacitation media. Capacitation was originally defined as the physiological membrane changes that take place inside the female reproductive tract and that enable spermatozoa to acquire fertilizing capacity (Chang 1951; Austin 1952). Since the development of *in vitro* fertilization (IVF), the definition of capacitation has been refined and is now considered to involve the consecutive activation of signalling pathways that induce physiological and biochemical modifications that prime the sperm cell for fertilization *in vitro* (Gervasi and Visconti, 2016). However, classical *in vitro* capacitation techniques (i.e., the inclusion of bicarbonate in the medium) achieve varying degrees of success in different species (Bailey, 2010). In horses, classical IVF (gamete co-incubation) is still a very poorly successful technique that has only ever yielded two foals, both born in France in the early 1990s (Palmer et al., 1991) and live foal production has not been reproduced. The fact that *in vitro* matured oocytes transferred to the oviduct of an inseminated mare yield a similar percentage of embryos to spontaneous ovulation (Hinrichs et al., 2002) and that *in vitro* treated sperm fail to penetrate both *in vivo* and *in vitro* matured oocytes (Tremoleda et al., 2003), suggests that the deficit in equine IVF may reside in an inability to adequately induce capacitation of stallion sperm under *in vitro* conditions.

The changes during capacitation render the sperm able to 1) bind to the oocyte extracellular matrix, the zona pellucida (ZP), and undergo an acrosome reaction (Saling et al., 1978; Saling and Storey, 1979; Topper et al., 1999); 2) acquire hyperactivation (Ho and Suarez, 2001); and 3) fuse with the oocyte plasma membrane (Evans and Florman, 2002). For capacitation to occur, the sperm must be held in environment (*in vivo* or *in vitro*) that contains bicarbonate (HCO_3_^−^), calcium (Ca^2+^), and albumin. These three factors are known to induce capacitation in many species, including mice (Visconti et al., 1995a; Visconti et al., 1995b), humans (Osheroff et al., 1999), and pigs (Flesch and Gadella, 2000). Besides inducing changes in membrane potential, these conditions alter cyclic adenosine monophosphate (cAMP) levels, intracellular pH (Leemans et al., 2019a), and intracellular Ca^2+^ (Gervasi and Visconti, 2016). Additionally, this capacitation-inducing environment leads to the removal of decapacitation factors from the surface of the sperm plasma membrane, leading to a “reorganization” of the plasma membrane lipid components and activation of several intracellular pathways (Gadella and Harrison, 2000; Flesch et al., 2001). Besides these three main elements, some species-specific factors for capacitation have also been identified. In cattle for example, heparin-like molecules such as glycosaminoglycans are essential for triggering capacitation (Parrish et al., 1988). However, no specific molecule has been identified as essential to triggering capacitation in stallion spermatozoa (Leemans et al., 2019b).

In the cauda epididymis, the HCO_3_^−^ concentration is very low (<1 mM), whereas when spermatozoa reach the fertilization site in the female genital tract the concentration is much higher (>15 mM) (Harrison, 1996). The rise in HCO_3_^−^ activates the sAC/cAMP/protein kinase A (PKA) pathway leading to an increase in membrane fluidity, and reorganization of the lipids in the plasma membrane, including the translocation of phosphatidylserine (PS) and phosphatidylethanolamine (PE) to the outer leaflet of the sperm plasma membrane (Flesch and Gadella, 2000; Gadella and Harrison, 2002). The translocation of these phospholipids during capacitation depends on the activation of PKA, which occurs after an increase in cAMP levels as a result of sAC activity (Visconti et al., 1995a). Phosphodiesterase (PDE) enzymes that metabolize cAMP to 5′-AMP (Nelson and Cox, 2004) also play a role in regulation of this process. As such, PDE inhibitors, such as caffeine, can maintain high levels of cAMP in the cell, thereby promoting capacitation, and spontaneous acrosome reaction with a consequent increase in sperm motility (Stephens et al., 2013). Another way to raise intracellular cAMP levels is by adding cell-permeable cAMP-analogues to the medium (Fraser, 1981; Visconti et al., 1995b; O’Flaherty et al., 2004).

Adenylyl cyclases (ACs) and their product, cAMP, have been implicated in several cellular signalling pathways in various cell types. In spermatozoa, the presence of sAC and transmembrane AC has primarily been studied in man and the mouse (Uguz et al., 1994; Harrison and Miller, 2000; Lefievre et al., 2000; Baxendale and Fraser, 2003; Spehr et al., 2004; Tardif et al., 2004; Wertheimer et al., 2013). In stallion sperm, there is limited data on the nature of the expressed ACs and their involvement in the specific steps of capacitation, the acrosome reaction, and hyperactivated motility. It is thought that bicarbonate, and thus bicarbonate-mediated activation of the sAC, is essential for cAMP upregulation and consequent initiation of capacitation in stallion sperm (Bromfield et al., 2014). However, recently the contribution of cAMP (generated by ACs) to hyperactivation in stallion spermatozoa has been questioned because cAMP upregulation was not detected under capacitating conditions (Leemans et al., 2019a). Based on these contrasting observations it is of vital interest to understand the extent to which ACs contribute to capacitation-related phenomena in stallion spermatozoa.

The current study aimed to investigate and clarify several aspects of stallion sperm capacitation as triggered by the presence of bicarbonate, calcium, and albumin. Specifically, membrane re-organization after the initiation of sperm capacitation was investigated using lipidomics, cryo-electron tomography, flow cytometry, and complementary biochemical strategies. To this end, PS and PE exposure in viable sperm were monitored by flow cytometry and PS exposure by live imaging. Finally, pharmacological assays were performed to determine which AC is responsible for the increase in membrane fluidity of stallion sperm under *in vitro* conditions.

## Materials and Methods

### Chemicals and Fluorescent Probes

All chemicals were purchased from Sigma-Aldrich (Zwijndrecht, Netherlands) unless otherwise stated. (±)-2-(1*H*-benzimidazol-2-ylthio) propanoicacid2-[(5-bromo-2 hydroxyphenyl)methylene] hydrazide (KH7) was from Sanbio (13243-10, Uden, Netherlands), 6-chloro-N4-cyclopropyl-N4-[(thiophen-3-yl)methyl]pyrimidine-2,4-diamine (LRE1; HY-100524) was obtained from MedChemExpress (NJ, United States). Annexin–V-FLUOS was purchased from Merck (11828681001, Darmstadt, Germany), lectin from *Arachis hypogea* (peanut) conjugated to Alexa Fluor^™^ 647 (PNA-AlexaFluor 647) was obtained from ThermoFisher Scientific (L32460; Waltham, MA, United States), and sodium; 3-[(2E)-2-[(E)-4-(1,3-dibutyl-4,6-dioxo-2-sulfanylidene-1,3-diazinan-5-ylidene) but-2-enylidene]-1,3-benzoxazol-3-yl] propane-1-sulfonate (merocyanine 540 abbreviated here to M540) was from Molecular Probes (M24571, Eugene, OR, United States). 4-(5-(4-Methylpiperazin-1-yl)-1H,1′H-[2,5′-bibenzo [d]imidazol]-2′-yl) phenol trihydrochloride (Hoechst 33258; 8861405) and 5.5‘,6.6‘-tetrachloro-1,1‘,3,3‘-tetraethylbenzimidazol-carbocyanine iodide (JC-1; T4069) were obtained from Sigma-Aldrich, and duramycin-cy5 was from Molecular Targeting Technologies, Inc., (D- 1002, West Chester, PS, United States). ADCY10 Polyclonal Antibody was purchased from Bioss Antibodies Inc., (bs-3916R, Woburn, MA, United States) and Goat anti-Rabbit IgG conjugated to Alexa Fluor^™^ 488 was from Life Technologies (A-11008, Bleiswijk, Netherlands). The semen extender was a commercial skim milk-based product (INRA 96) purchased from IVM technologies (016441, l’Aigle, France).

### Media for Sperm Incubation

The basic variant of Tyrode’s medium (Tyr_Control_) consisted of 111 mM NaCl, 20 mM HEPES, 5 mM glucose, 3.1 mM KCl, 0.4 mM MgSO_4_, 0.3 mM KH_2_PO_4_, 100 µg/ml gentamycin sulfate, 1.0 mM sodium pyruvate, 21.7 mM sodium lactate. In the bicarbonate containing variant (Tyr_Bic_) a defined amount of NaCl was replaced by 30 mM of NaHCO_3_. The pH was adjusted to 7.40 ± 0.05 at room temperature with NaOH or HCl and the osmolality to 300 ± 5 mOsmol/kg. All media were passed through a polyethersulfone syringe filter (PES membrane, pore size 0.22 µm; Merck Millipore, Amsterdam, Netherlands) for sterile filtration. Both media contained 1 mg/ml of bovine serum albumin (BSA; A6002, Sigma-Aldrich) and 2 mM of Ca^2+^ supplemented as CaCl_2_. The bicarbonate containing medium (Tyr_Bic_) and its variants were kept in an incubator with 5% CO_2_ and 100% humidity at 37°C for at least 24 h for equilibration prior to experimentation. Incubations of spermatozoa in bicarbonate containing media took place in the same incubator used for equilibration. Incubations of spermatozoa in control medium (Tyr_Control_) were carried out in a metal heating block at 37°C.

### Semen Collection and Dilution

Semen was collected using an artificial vagina (Hanover model) from stallions attending the Faculty of Veterinary Medicine at Utrecht University for routine breeding soundness examination, or from stallions located at nearby horse farms (Stal Schep and Stal van Vliet) with the written consent of the owners. After collection, semen was filtered through gauze to remove the gel fraction and gross debris. A smear of raw semen with Aniline Blue-Eosin was prepared to assess sperm morphology. Sample concentration was measured with a Bürker Türk haemocytometer and ejaculates were diluted in INRA 96^®^ to a concentration of 30 × 10^6^ spermatozoa/mL. Motility was checked objectively using a computer-assisted semen analysis (CASA) system (SpermVision 3.5, Minitüb, Tiefenbach, Germany) as described by Brogan et al. (2015). Only samples with greater than or equal to 70% (total) motile sperm in the diluted semen were used for experiments. Diluted semen was kept at room temperature until further processing. For each experiment, semen from a minimum of three different stallions was used, with the exact number of replicates stated in each figure caption.

### Semen Preparation for Experiments

Density gradient centrifugation was performed to separate the spermatozoa from the semen extender and seminal plasma prior to experimentation. Diluted semen (6 ml) was layered on top of a discontinuous gradient consisting of 2 ml of isotonic 70% Percoll^®^-saline solution and 4 ml of isotonic 35% Percoll^®^-saline solution in a 15-ml centrifugation tube, as described by Harrison et al. (1993). Tubes were centrifuged for 20 min at room temperature; 10 min at 300 g followed by 10 min at 750 g, without stopping in between. After centrifugation, the supernatant was removed and the remaining pellet was resuspended in 1 ml of Tyr_Control_ without CaCl_2_ and BSA. The sperm concentration was adjusted to 30 × 10^6^ sperm/mL, unless otherwise stated. The sperm suspension was used within 30 min of preparation.

### Flow Cytometry Analysis

#### Flow Cytometer

A FACS Canto II flow cytometer (BD Biosciences, Breda, Netherlands) was used to assess membrane changes in stallion spermatozoa. The machine was equipped with laser lines at 405 nm (30 mW), 488 nm (20 mW), and 633 nm (17 mW). A gate on forward and side scatter characteristics identified the single sperm population. For each sample, data from 10,000 individual spermatozoa were acquired at medium speed (35 µL ± 5 µL/min). Signals for the fluorescent dyes were collected through a 450/50 nm filter (Hoechst 33258), 530/30 nm filter (JC-1 monomers, Annexin-V-FLUOS), 585/42 nm (JC-1 aggregates, M540), and a 660/20 nm (PNA-Alexa Fluor^™^ 647, Duramycin-Cy5). Data were analyzed using FCS Express (version 3 and 7, De Novo Software, Glendale, CA, United States). Spectral overlap between dyes was compensated post acquisition.

#### General Stimulation of cAMP-Dependent Pathways

Where indicated, a final concentration of 1 mM caffeine (2,760, Sigma-Aldrich) and/or 1 mM N^6^,2′-O-Dibutyryladenosine 3′,5′-cyclic monophosphate sodium salt (db-cAMP; D0260, Sigma-Aldrich) were added to Tyr_Control_ and Tyr_Bic_ prior to experimentation.

#### Assessment of Viability, Acrosome Integrity, and Membrane Fluidity

Ten microliter Percoll-washed sperm aliquots were added to FACS tubes containing 490 µL of either Tyr_Control_ or Tyr_Bic_. All media contained Hoechst 33258 and PNA-AlexaFluor 647. Samples were then incubated for 15, 30, and 60 min. Fifteen minutes before measurements took place, 2 µL M540 (stock solution: 750 mM in DMSO) was added to the tube and the incubation continued. FACS tubes were capped prior to removal from the incubator and transported in a metal heating block at 37°C to the flow cytometer. The transport time was less than 30 s. Before analysis on the flow cytometer, samples were briefly vortexed.

#### Assessment of PS or PE Exposure

PS exposure was detected with the probe Annexin-V-FLUOS. Spermatozoa were incubated in 500 µL Tyr_Control_ or Tyr_Bic_ with Hoechst 33258 and PNA-AlexaFluor^™^ 647. Fifteen minutes before a measurement, 100 µL of the tube’s content was transferred to a new, prewarmed tube and 2 µL of Annexin-V-FLUOS was added. The Annexin-V staining was carried out in the presence or absence of 0.2 µL M540. PE exposure was detected with duramycin-Cy5. Samples were incubated in 500 µL Tyr_Control_ or Tyr_Bic_ with Hoechst 33258 and PNA-AlexaFluor488 (stock solution: 0.25 mg/ml in aqua dest). Five minutes before a measurement, 2 µL of duramycin-Cy5 (stock solution: 0.5 mg/ml in 1% DMSO in aqua dest) was added.

#### Assessment of Mitochondrial Transmembrane Potential

Spermatozoa were incubated in 500 µL Tyr_Control_ or Tyr_Bic_ with Hoechst 33258 and PNA-AlexaFluor 647. Fifteen minutes before a measurement was due, 2 µM JC-1 (stock solution: 250 µM in DMSO) was added to a tube and the content briefly mixed.

#### Inhibition of sAC with KH7 or LRE1

Three different concentrations of either KH7 or LRE1 (10, 60, and 120 µM) were tested in Tyr_Control_ and Tyr_Bic_ media. Concentrations were based on previous published work on mouse and stallion spermatozoa (Hess et al., 2005; McPartlin and Visconti, 2011). These inhibitors were added to the sperm suspension directly after Percoll centrifugation. In these experiments Tyr_Control_ and Tyr_Bic_ were also supplemented from the beginning with the respective KH7 or LRE1 concentration. Samples processed in presence of DMSO served as solvent controls. Tubes were incubated for 15, 30, and 60 min. All media contained Hoechst 33258 and PNA-AlexaFluor 647. Merocyanine 540 or JC-1 were added 15 min prior to the measurements on the flow cytometer.

### Immunolabelling of Soluble Adenylyl Cyclase

#### Sperm Preparation for Immunocytochemistry and Immunoblotting

For this experiment, stallion and boar spermatozoa (from AIM Varkens KI Netherlands) were used to compare the subcellular localization of sAC in the sperm cell. Boar spermatozoa were used as a control because these cells have previously been reported to have high sAC activity (Leemans et al., 2019a). This experiment used one ejaculate from three different animals from each species (*n* = 3 biological replicates). For both species, the spermatozoa were separated from the semen extender and seminal plasma by density gradient centrifugation. Diluted semen (9 ml) was layered on top of 3 ml of 35% Percoll^®^-saline in a 15-ml centrifugation tube and centrifuged at 750 x *g* for 10 min at room temperature. After centrifugation, the supernatant was removed. The remaining pellet was resuspended in Dulbecco’s PBS (DPBS). For immunolocalization experiments, 500 µL of the sperm preparation was incubated with 500 µL of 4% paraformaldehyde for 15 min at room temperature for fixation. After fixation, the samples were centrifuged for 5 min at 600 x *g*. The supernatant was removed, the pellet diluted in DPBS and another centrifugation was performed.

#### Immunofluorescent Labelling of sAC

Next, the samples were settled onto Superfrost^™^ (Thermo Fisher) glass slides for 1 h at room temperature, after which slides were washed once before the addition of a 0.2% Triton X-100 solution for cell permeabilization (10 min at room temperature). Cells were then blocked with 3% BSA/PBS for 1 h and washed once in DPBS. Anti-ADCY10 primary antibody (Bioss Antibodies; bs_3916R) was applied (final concentration 10 µg/ml) and slides were incubated overnight and then washed twice with DPBS. Next, the secondary antibody [goat anti-mouse IgG (H + L), Alexa Fluor 488] was added and slides were incubated for 1 h at room temperature. Hoechst 33342 and PNA-AlexaFluor647 were also included to stain the nuclei and the acrosome of the sperm, respectively. After the incubation, slides were washed with PBS and later covered with 5 µL of Vectashield (Vector Laboratories, California, United States) and a coverslip that was sealed on with nail varnish. Vectashield was added to prevent the fluorescence from bleaching. For cell imaging, a laser scanning confocal microscope (LEICA SPE II DMI 4000, Leica Microsystems, Wetzlar, Germany) was used. On the LEICA SPE II DMI 4000, Hoechst33342 which labels all DNA was excited with the 405 nm laser and the secondary antibody (conjugated to Alexa Fluor 488) for detection of the ADCY10, was excited with the 488 nm laser. PNA-AlexaFluor647 was excited using a 633 nm laser for acrosome detection.

#### Immunoblot Detection of sAC

To prepare samples for immunoblotting, DPBS was removed *via* centrifugation, and replaced with 500 µL of sodium-dodecylsulphate (SDS) extraction buffer (0.375 M Tris pH 6.8, 2% w/v SDS, 10% w/v sucrose, protease inhibitor cocktail). Samples were boiled at 100°C for 5 min and insoluble material was removed by centrifugation (17,000 *g*, 10 min); soluble protein remaining in the supernatant was quantified using a BCA protein assay kit (ThermoFisher Scientific). Equivalent amounts of protein lysates (10 μg for both boar and stallion samples) were boiled in SDS-polyacrylamide gel electrophoresis (PAGE) sample buffer (2% v/v beta-mercapto-ethanol, 2% w/v SDS, and 10% w/v sucrose in 0.375 M Tris, pH 6.8, with bromophenol blue) at 100°C for 5 min, prior to being resolved by SDS-PAGE (150 V, 1 h) and transferred to nitrocellulose membranes (350 mA, 1.5 h). Membranes were then blocked in 3% BSA diluted in Tris-buffered saline (TBS) supplemented with 0.1% (v/v) Tween-20 (TBST), and then incubated with anti-ADCY10 (Bioss Antibodies; bs_3916R) diluted in 1% BSA/TBST (final concentration 2 µg/ml). Immunoblots were washed 3 × 10 min at room temperature with TBST before being probed with appropriate horse-radish peroxidase (HRP)-conjugated secondary antibodies. After three further washes, labelled proteins were detected using an enhanced chemiluminescence kit (ECL-detection kit; Supersignal West Pico, Pierce, Rockford IL, United States). All immunoblots were re-probed with anti-GAPDH antibodies and appropriate secondary antibodies to check for equivalent protein loading.

### Live Imaging of Annexin-V Staining Patterns

A live imaging approach was used to demonstrate whether Annexin-V staining was present in viable stallion sperm under capacitating conditions. To this end, 10 µL of Percoll-washed sperm (120 × 10^6^ sperm/mL) was incubated in 500 µL of either Tyr_Bic_ or Tyr_Bic_ supplemented with 1 mM caffeine. Previous experiments indicated that these conditions yielded the highest abundance of Annexin-V positive spermatozoa. Both media contained Hoechst 33258 and PNA-AlexaFluor647, as described for flow cytometry. Samples were evaluated after total incubation times of 30 and 60 min, respectively. 15 min before measurements took place, 100 µL of the sample was transferred to a pre-warmed Eppendorf tube, and 2 µL of Annexin-V FLUOS was added. Live imaging was performed on a NIKON STORM/A1Rsi/TIRF microscope (Nikon, NY, United States), with a preheated stage at 37 °C. After incubation, the samples were centrifuged for 2 min at 1000 x *g* and then resuspended in 10 µL of the respective medium. Next, a 1 µL droplet was placed in a FluoroDish (FD35-100, World Precision Instruments, Friedberg, Germany) and covered with a round coverslip (diameter: 8 mm). Pre-warmed mineral oil was placed around the coverslip to prevent the sample from drying. The autofocus function of the microscope was used to locate the imaging plane. General imaging settings in the acquisition software were: 40x objective, scan speed ½; image size 1,024 × 1,024 pixels, pinhole 5.0 and zoom 1. At least five large scans were performed in a 2 × 2 panel series with laser lines at 405 nm, 488 nm, and 647 nm and corresponding detection channels for the dyes (Hoechst 33258, Annexin-V-FLUOS, AlexaFluor 647, DIC). Images were analyzed with NIS Elements Viewer software (Nikon, NY, United States). An automated detection of the different fluorescent signals in each cell was performed. Subsequently, the staining patterns were validated visually and the location of Annexin V staining (PS exposure) was determined. At least 200 viable cells were scored for each medium.

### Sperm Sorting for Lipidomics and Cryo-Electron Tomography

Stallion spermatozoa were sorted on a FACS Influx (Becton Dickinson, San Jose, Canada). A total of 4 × 10^7^ spermatozoa were incubated for 60 min in either 0.5 ml Tyr_Control_ or Tyr_Bic_ media supplemented with 2 µL Hoechst 33258. M540 was added 15 min before sorting. Hoechst 33258 was excited with a 405 nm Laser. Emission was captured with a 460/50 nm filter. M540 was excited with a 561 nm laser, and emission was captured with a 585/42 nm filter. Spermatozoa were analyzed at a rate of between 8,000 and 10,000 events per second. Only events with forward and side scatter characteristics of single spermatozoa were considered for further analysis. During sorting, the sample-input tube on the FACS Influx was kept at 38°C to maintain the sample’s temperature during the entire sorting procedure. Phosphate-buffered saline served as sheath fluid. Two subpopulations were sorted: 1) viable spermatozoa (Hoechst 33258 negative) with low membrane fluidity (M540 fluorescence low) from Tyr_Control_, and 2) viable spermatozoa with high membrane fluidity (M540 fluorescence high) from Tyr_Bic_. A total of 250,000 spermatozoa from a specific subpopulation were sorted into a single tube. The sorting time per tube ranged from 8 min to 15 min. Immediately after sorting, the tube was centrifuged at 11,000 x *g* for 10 min and the supernatant discarded.

### Cryo-Electron Tomography

Pellets of sorted spermatozoa were diluted to ∼3 × 10^6^ cells/mL in phosphate buffered saline. Approximately 3 µL of cell suspension was applied to glow-discharged Quantifoil R 2/1 200-mesh holey carbon grids. Approximately 1 µL of BSA-gold (Aurion, Wageningen, Netherlands) was added, after which grids were blotted manually from the back for 3–4 s and immediately plunged into a 37% liquid ethane/propane mix cooled to liquid nitrogen temperature. Grids were stored under liquid nitrogen until imaging. Imaging was performed on a Talos Arctica (ThermoFisher) operated at 200 kV and equipped with a post-column energy filter (Gatan) in zero-loss imaging mode with a 20-eV energy-selecting slit. All images were recorded on a ∼ 4k × 4k K2 Summit direct electron detector (Gatan) in counting mode with dose-fractionation. Tilt series were collected with SerialEM, using a grouped dose-symmetric tilt scheme covering an angular range of ± 56° in 2° increments. Tilt series were acquired with a Volta phase plate (VPP) at a target defocus of −0.75 µm and with a pixel size of 3.514 Å. The total dose was limited to <100 e^−^/Å^2^. Frames were aligned using Motioncor2 1.2.1. Tomograms were reconstructed in IMOD 4.10.25 using weighted back-projection. Contrast Transfer Function (CTF) correction was not performed because tilt series were acquired close to focus with the VPP. For segmentation and presentation, 6x-binned tomograms were reconstructed with a simultaneous iterative reconstruction technique (SIRT)-like filter corresponding to 20 iterations. Membrane thickness and intermembrane distances from at least five selected tomograms for each sample group were measured in Fiji (Schindelin et al., 2012). For all selected tomograms, ten measurements were performed for each compartment (plasma membrane, intermembrane distance and outer acrosomal membrane when possible). Measurement locations were spaced by at least 100 nm.

### Lipid Analysis

#### Lipid Extraction From Stallion Spermatozoa

After pelleting the sorted spermatozoa (6 biological replicates and two technical replicates), the supernatant was discarded. The cell pellets were held for 10 min in a box with constant N_2_ gas supply to eliminate oxygen and then stored at −20°C. For mass spectrometry the technical replicates were pooled together to form a total cell pellet of 5,00,000 sperm cells for each biological sample (to aid in the detection of poorly abundant lipids) and these cell samples (*n* = 6) were then transferred to glass vials for lipid extraction. A 1:1 chloroform/methanol (C/M) solution containing three reference lipids: 0.5 µM sitosterol; 0.05 µM 3-keto cholesterol and 0.5 µM 1,2-dimyristoyl-sn-glycero-3-phosphocholine (DMPC) was added to each sample for a total volume of 200 µL. Sperm lipids were extracted in this solution by incubating samples for 30 min at room temperature with gentle mixing. Samples were then centrifuged at 2000 x *g* for 10 min and supernatants containing the organic phase were transferred to new mass spectrometry grade glass inserts and vials. Samples were then dried under nitrogen and resuspended in 30 µL of 1:1 C/M prior to use for mass spectrometry. Remaining 1:1 C/M with the reference lipids was used as a control.

#### Detection of Cholesterol and Desmosterol

Extracted lipids were loaded on a C8-column (2.6 μm Kinetex C8 100 Å, 150 × 3.0 mm, Phenomenex, Torrance, CA, United States) maintained at 40°C and eluted at a flow rate of 0.6 ml/min. A gradient elution was performed from methanol/water (1/1; v/v) to methanol/iso-propanol (4/1; v/v) in 2 min, followed by isocratic elution with the latter solvent for an additional 7 min. A 1 min re-equilibration time was used between runs. The column outlet of the LC (Dionex HPG-3200RS UPLC; Thermo Fisher Scientific, Waltham, MA, United States) was connected to the atmospheric pressure chemical ionization source of an LTQ-XL mass spectrometer (Thermo Fisher Scientific). Full scan spectra were collected in positive ionization mode in the range from 200 to 1100Da.

#### Detection of Phospholipids

Lipid extracts in 1:1 C/M were injected (5 µL in triplicate) onto a hydrophilic interaction liquid chromatography (HILIC) column (2.6 μm HILIC 100 Å, 50 × 4.6 mm, Phenomenex, Torrance, CA, United States). Lipid classes were separated by gradient elution on an Infinity II 1290 UPLC (Agilent, Santa Clara, CA, United States) at a constant flow rate of 1 ml/min. Acetonitrile/acetone (9:1, v/v) was used as solvent A and Solvent B consisted of a mixture of acetonitrile/H_2_O (7:3, v/v) with 10 mM ammonium formate. Both solvents contained 0.1% formic acid. The gradient used was (time in min, %B): (0, 0), (1, 50), (3, 50), (3.1, 100), (4, 100). The column flow was connected to a heated electrospray ionization (H-ESI) source of an Orbitrap Fusion mass spectrometer (ThermoScientific) operated at –3,600 V in the negative ionization mode. Temperatures for the vaporizer and ion transfer tube were 275°C and 380°C, respectively. Full scan MS1 measurements in the mass range from 420 to 1150 u were collected in the Orbitrap at a resolution of 1,20,000. Data-dependent MS2 experiments were performed in parallel to the Orbitrap MS1 scanning by fragmentation through higher-energy collisional dissociation, set at 30 V, using the dual-stage linear ion trap to generate up to 30 spectra per second.

#### Data Analysis

Acquired raw datafiles were converted to mzML files by msConvert (part of ProteoWizard v3.0.913) and processed with the R package xcms v2.99.3. Annotation of lipids was performed by matching measured MS1 *m/z* values with theoretical *m/z* values as described in Molenaar et al. (2019). Peak intensities of the annotated lipids were deisotoped and corrected for recovery from the internal standard. Resulting data are included in Supplementary File S1.

### Statistics

Data were analyzed using the Statistical Analysis System software (SAS^®^, version 9.4; SAS Inst. Inc., Cary, NC, United States). Parameters were tested for normal distribution using the Shapiro-Wilk test. Where applicable, a multivariate analysis of variance (ANOVA) for repeated measurements was performed. Comparisons between individual treatments or time points were carried out using Student’s t-test for paired observations. All data are presented as mean ± standard deviation (SD). Differences were considered significant when *p* ≤ 0.05.

## Results

### Bicarbonate Induces an Increase in Membrane Fluidity in Viable Sperm

**FIGURE 1:**
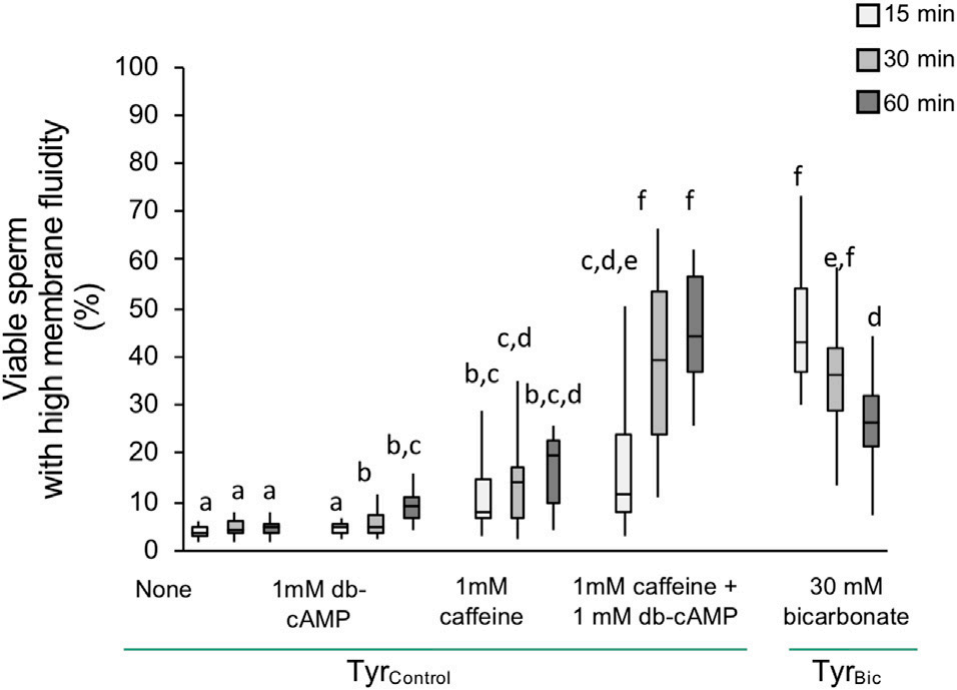
An increase in plasma membrane fluidity in viable stallion spermatozoa is dependent on cAMP signalling. Spermatozoa were in incubated in Tyrodes (Tyr) medium either in the presence of 30 mM bicarbonate (Tyr_Bic_) or its absence (Tyr_Control_). An increase in plasma membrane fluidity, i.e. increased merocyanine 540 staining, in viable (Hoechst 33258 negative) spermatozoa was induced by bicarbonate within 15 min. A similar, but delayed, increase was evoked by 1 mM caffeine and 1 mM bd-cAMP (*n* = 8 stallions; *p* < 0.05).

Initial experiments were conducted to confirm that the experimental conditions would stimulate an increase in membrane fluidity (as previously demonstrated by Rathi et al. (2001)). Our results indicated that incubating stallion spermatozoa in Tyr_Bic_ medium resulted in a significantly increased population of viable spermatozoa with high membrane fluidity after a 15 min incubation time (Figure 1). In the absence of bicarbonate no change was observed. The combination of a cAMP analogue (db-cAMP) and a PDE inhibitor (caffeine) was able to mimic the bicarbonate effect and induced an equally large population of viable sperm with high membrane fluidity (Figure 1; Supplementary Figure S1I). Nonetheless, the response was slower than for Tyr_Bic_ and it took at least 30 min before this population was detectable (Figure 1). It is thought that bicarbonate is essential for cAMP upregulation and in other eutherian mammals this response is mediated by adenylyl cyclase. Indeed, in spermatozoa, the presence of soluble adenylyl cyclase (sAC) has primarily been studied in human and mouse sperm (Uguz et al., 1994; Harrison and Miller, 2000; Lefievre et al., 2000; Baxendale and Fraser, 2003; Spehr et al., 2004; Tardif et al., 2004; Wertheimer et al., 2013). In stallion sperm, there is limited available data regarding the nature of sACs and its involvement in the specific steps of capacitation. Thus, the following studies were designed to determine whether the impact of bicarbonate on stallion sperm membrane fluidity is mediated by sAC.

### Detection of sAC in Stallion and Boar Sperm

**FIGURE 2:**
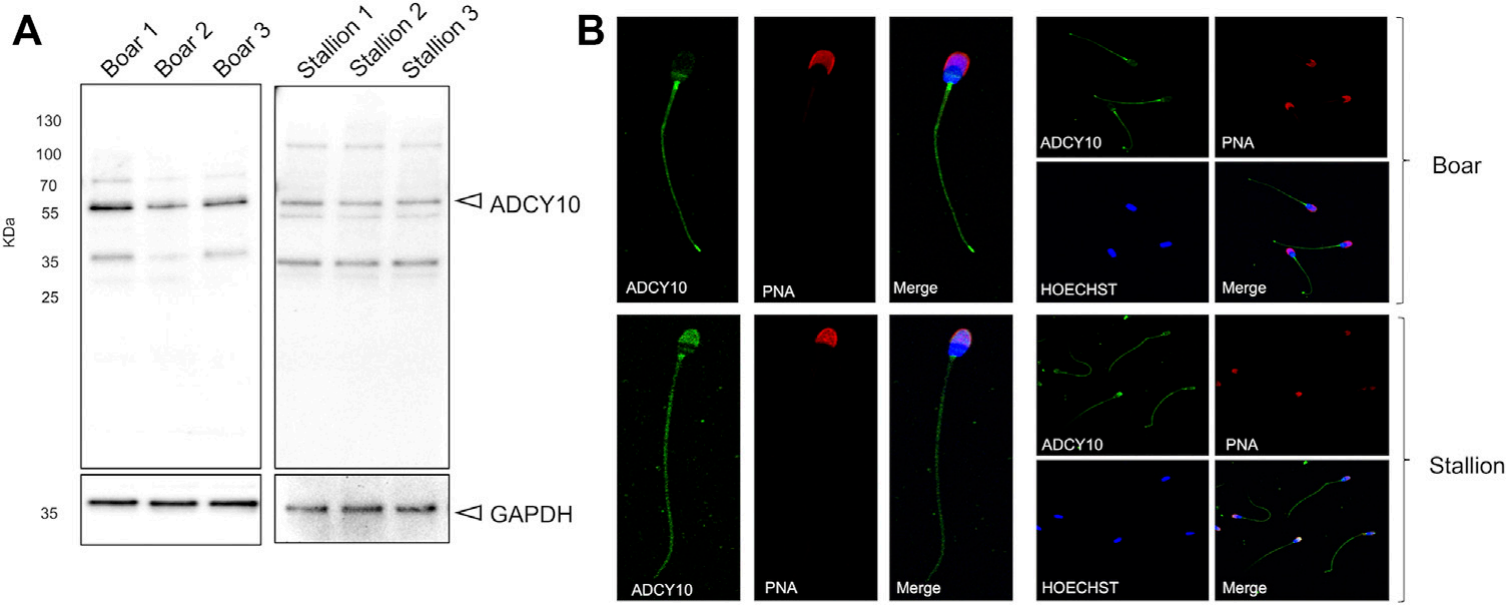
Detection and localization of ADCY10 in stallion and boar spermatozoa. Native semen samples were processed for immunoblotting and immunofluorescence with a polyclonal antibody against ADCY10. **(A)** Immunoblot of anti-ADCY10 in boar semen samples (*n* = 3 boars) and stallion semen samples (*n* = 3 stallions). GAPDH was used as a loading control. **(B)** Representative images for the localization of ADCY10 in boar spermatozoa and stallion spermatozoa (*n* = 3 boars; *n* = 3 stallions). Samples were counterstained with PNA-AlexaFluor^™^ 647 (to visualize sperm acrosomes) and Hoechst 33342 (to visualize the nucleus).

To investigate the presence and localization of sAC in stallion spermatozoa, immunoblotting and immunofluorescence with an anti-ADCY10 antibody was performed. The results were compared sAC localization in boar sperm, where it is known to have high activity (Leemans et al., 2019b), and shares 87.03% sequence similarity to equine ADCY10. Immunoblotting demonstrated that the sAC is present in both boar and stallion spermatozoa as an immunoreactive band at approximately 55 kDa (Figure 2A). This is the predicted size of the testis-specific form of sAC. In both species, some additional bands were detected at approx. 35 and 50 kDa. Immunofluorescent labelling of sAC revealed that this adenylyl cyclase is distributed along the tail of spermatozoa from both species, with bright labelling noted in the endpiece of the tail. However, a higher signal intensity for both species was noted in the neck region. In the sperm head, species-specific staining patterns were observed with sAC localized over the whole acrosomal area in stallion spermatozoa, whereas the sAC signal was limited to a distinct band across the post-equatorial region of boar spermatozoa (Figure 2B). Secondary controls where Anti-ADCY10 was omitted revealed no non-specific fluorescence (Supplementary Figure S1).

### Inhibition of sAC Prevents an Increase in Membrane Fluidity

**FIGURE 3:**
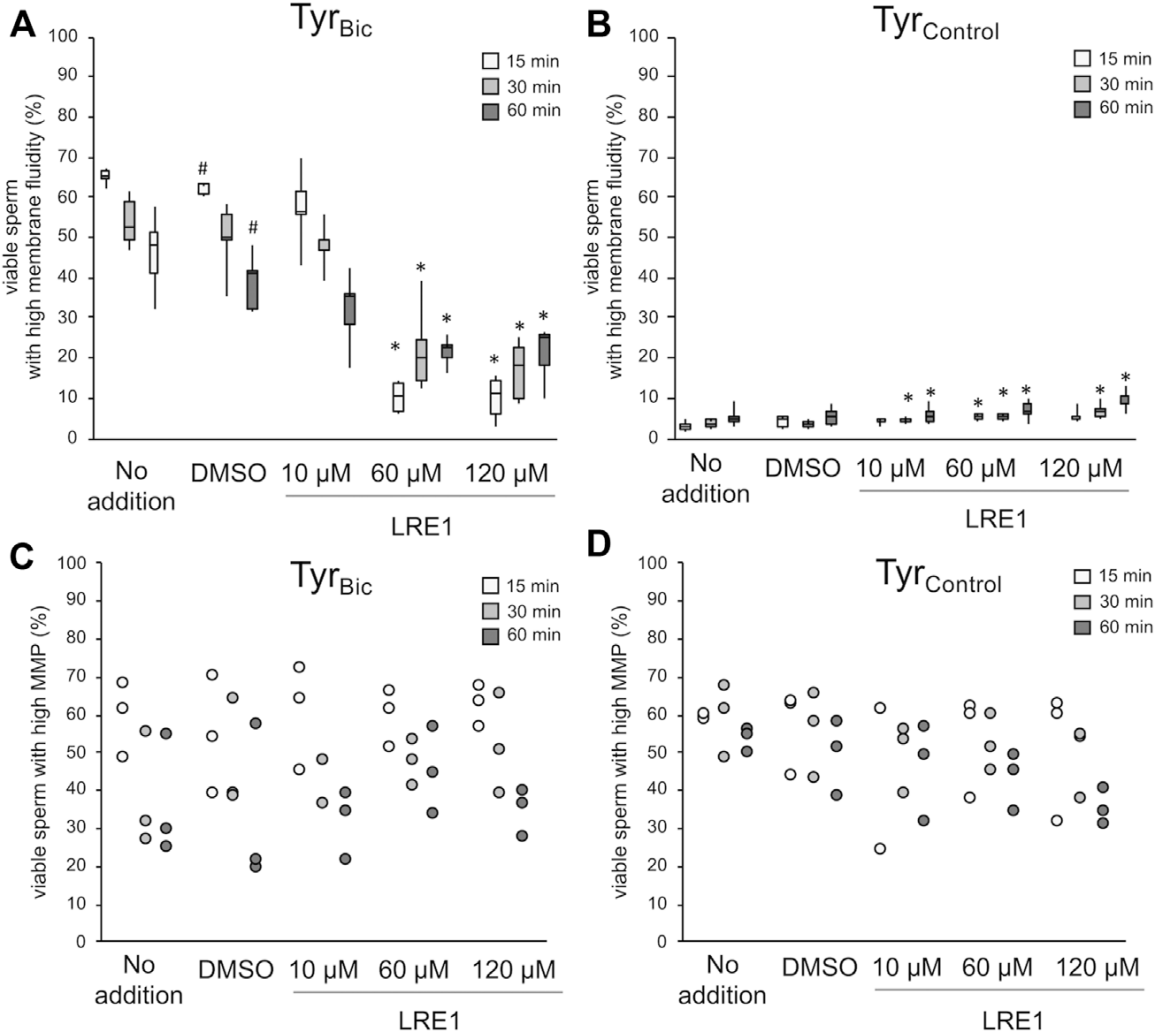
LRE1 blocks sAC activity with no off-target effects on mitochondrial membrane potential. Spermatozoa were incubated either in presence of 30 mM bicarbonate (Tyr_Bic_) or its absence (Tyr_Control_). Media contained either no further additions, DMSO (solvent control), or an increasing concentration of LRE1 to directly block sAC activity. An increase in plasma membrane fluidity, i.e., increased merocyanine 540 staining, in viable (Hoechst 33258 negative) sperm was monitored in absence or presence of LRE1 [**(A,B)**; *n* = 6 stallions]. A hash symbol (#) indicates significant differences between DMSO exposed samples and samples without any additions (*p* < 0.05). An asterisk (*) indicates significant differences between DMSO exposed samples and samples treated with LRE1 (*p* < 0.05). In a subset of samples, the percentage of viable sperm with high mitochondrial transmembrane potential (MMP) was monitored using the probe JC-1 [**(C,D)**; *n* = 3 stallions].

Following confirmation that sAC was present in stallion spermatozoa, the effect of sAC inhibition was then assessed in this species. Indirect inhibition of sAC with the inhibitor KH7 in the presence of bicarbonate (Tyr_Bic_) appeared to be effective in a large proportion of viable spermatozoa at concentrations of 60 and 120 µM (Supplementary Figure S2A). However, off-target effects were also observed, with KH7 at 60 and 120 µM resulting in an increase in viable sperm populations with high membrane fluidity in Tyr_Control_ but abolishing the high mitochondrial transmembrane potential in virtually all spermatozoa (Supplementary Figures S2B–D). Further observations confirmed that spermatozoa stopped tail beating within 1 min after exposure to KH7 at all concentrations. This is in line with a previous report suggesting that KH7 acts as a mitochondrial uncoupler (Jakobsen et al., 2018). Given these challenges, a direct inhibitor of sAC, LRE1, which specifically competes with the bicarbonate binding site of sAC (Ramos-Espiritu et al., 2016), was used in equivalent experiments. Using LRE1, the expected increase in membrane fluidity could be prevented in most of the viable spermatozoa after incubation for 15 min in the presence of 30 mM bicarbonate (Tyr_Bic_; Figure 3A). The strength of the inhibition lessened over time, but was still significant after 60 min of incubation (Figure 3A). A small, but significant increase in the percentage of viable spermatozoa with high membrane fluidity was noted in the absence of bicarbonate (Tyr_Control_). However, this population stayed below 10% (Figure 3B). In contrast to the observations for KH7, there were no adverse effects of LRE1 in Tyr_Bic_ or Tyr_Control_ on the percentage of spermatozoa with high mitochondrial membrane potential, and the cells remained motile (Figures 3C,D).

### Cryo-EM Reveals Membrane Reorganization in the Acrosomal Area of Viable Sperm with High Membrane Fluidity

**FIGURE 4:**
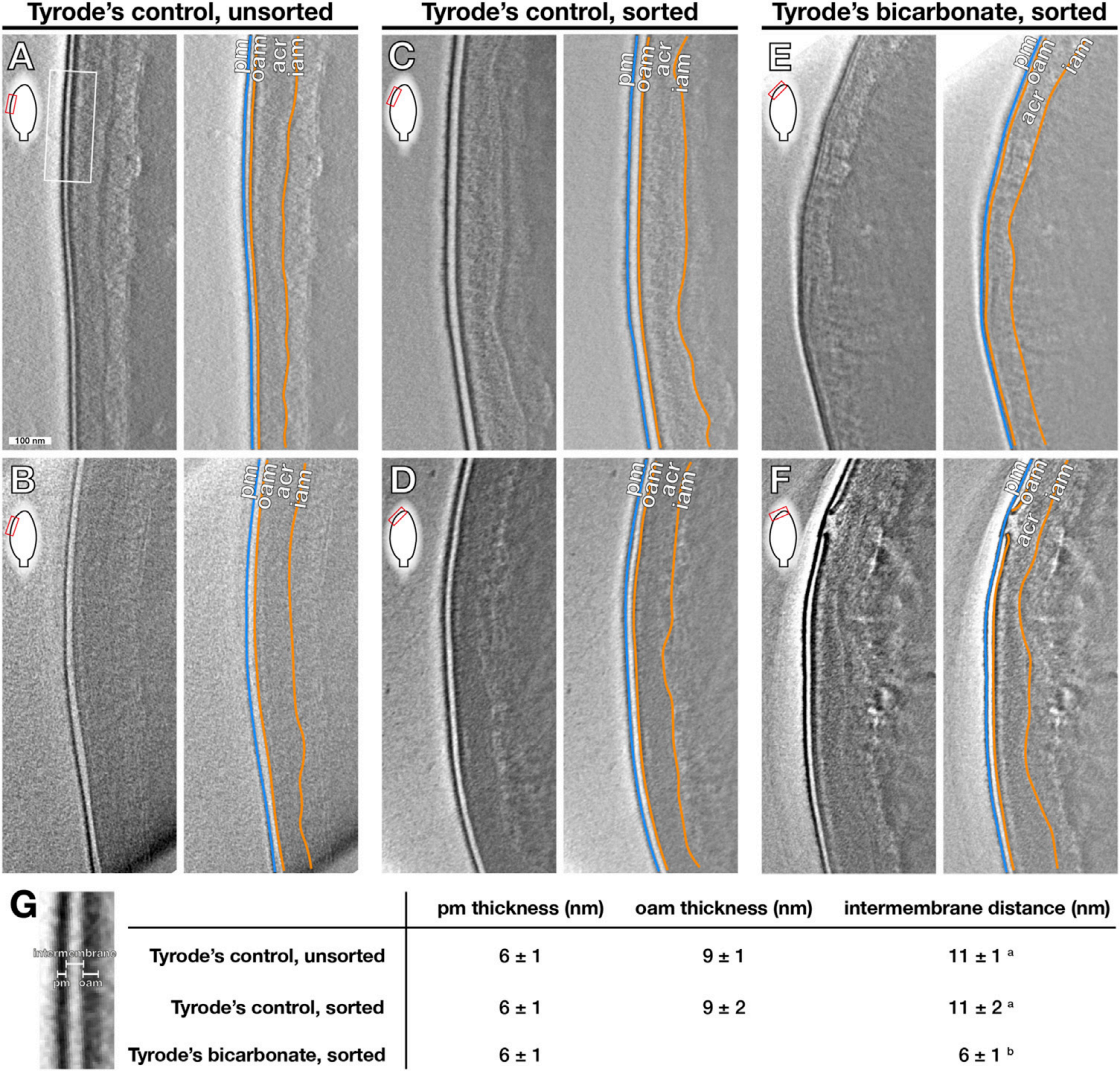
Sperm membrane reorganization detected by Cryo-ET after incubation with bicarbonate. Spermatozoa were incubated either in the presence of 30 mM bicarbonate (Tyr_Bic_) or its absence (Tyr_Control_) for 60 min and then FACS-sorted to inspect membrane reorganization using cryo-electron tomography (cryo-ET). To mark structures identified by cryo-ET, the membranes are colour coded to indicate the plasma membrane (PM) (blue), outer acrosomal membrane (OAM), and inner acrosomal membrane (IAM) (orange) that surround the acrosomal content. Red boxes indicate the approximate region of the sperm head featured in the frames. Unsorted spermatozoa in Tyr_Control_ typically demonstrated an intact PM and OAM that were smooth and ran parallel to each other along the sperm head **(A,B)**. This was also observed in a majority of FACS-sorted viable, “low fluidity” cells in TyrControl **(C,D)** (*n* = 3 stallions). However, sorted viable, “high fluidity” sperm cells incubated in Tyr_Bic_ mostly showed evidence of membrane remodelling and destabilization, including closer approximation of the PM and OAM **(E)** in particular at **(F)** points of apparent rupture of the OAM **(F)**, leading to a decrease in apparent intermembrane distance **(G)** (*n* = 3 stallions).

To visualize structural changes associated with high membrane fluidity, we imaged whole, unfixed, unstained stallion sperm using cryo-electron tomography (cryo-ET). Cryo-ET yields three-dimensional reconstructions of subcellular structures within the context of fully-hydrated cells, avoiding artefacts from dehydration and chemical fixation. To establish baseline membrane morphology, we imaged non-sorted spermatozoa that had been incubated for 60 min in Tyr_Control_ (Figures 4A,B). In these non-capacitated cells, the plasma membrane (PM) and outer acrosomal membrane (OAM) were smooth and parallel (10/12 tomograms, each from a different cell, from two stallions), with a regular intermembrane distance of ∼11 ± 2 nm. To directly correlate structural changes to membrane fluidity, we imaged flow-sorted viable sperm with either low or high M540 staining. The majority of sorted, viable sperm with low membrane fluidity were similar to non-sorted control cells, with the PM and OAM both intact, smooth, and running parallel to each other (13/19 tomograms, each from a different cell, from three stallions) (Figures 4C,D). In contrast, most of the sorted, viable spermatozoa with high membrane fluidity showed evidence of membrane destabilization (23/26 tomograms, each from a different cell, from three stallions). A range of phenotypes could be distinguished. Some cells showed a clear approximation of the PM and OAM (Figure 4E), in particular at sites where the OAM becomes discontinuous and bends upwards towards the OM (Figure 4F) (6 tomograms, each from a different cell). This was reflected by a reduced average intermembrane distance (Figure 4G). Finally, in some spermatozoa the presence of membrane vesicles was observed in regions close to sites of membrane disruption (Supplementary Figure S1).

### Membrane Reorganization is Not Accompanied by a Change in Sperm Lipid Composition

**FIGURE 5:**
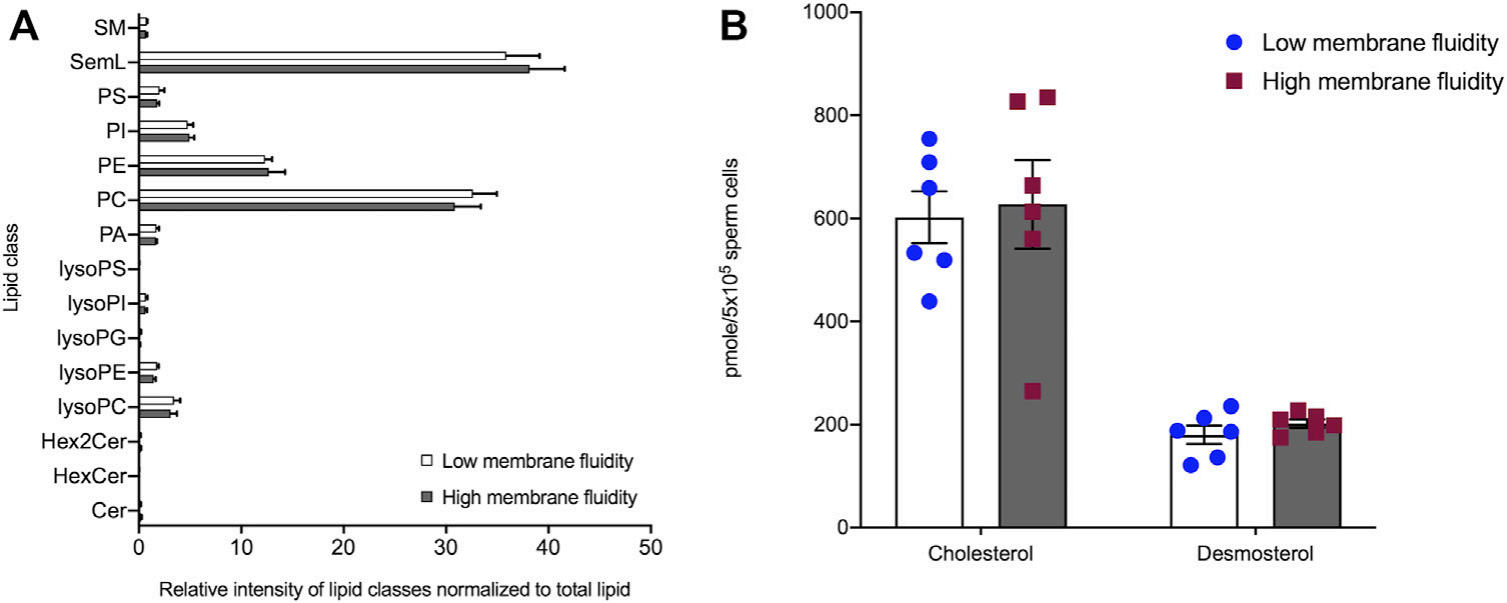
Lipidomic analysis of stallion sperm populations with low- and high-membrane fluidity selected by FACS-sorting. Stallion spermatozoa were incubated for 60 min in either Tyr_Control_ or Tyr_BIC_ medium supplemented with Hoechst 33258. Fifteen minutes prior to FACS sorting M540 was added to each population. Using a FACS Influx, stallion sperm cells were sorted into two subpopulations; viable sperm with low membrane fluidity (Hoechst negative, M540 “low”); and viable sperm with high membrane fluidity (Hoechst negative, M540 “high”). Samples were then processed for either phospholipid and glycolipid analysis or quantitation of cholesterol and desmosterol. Phospholipid and glycolipid analysis revealed 15 classes of phospholipids present in stallion spermatozoa **(A)** with a relative abundance of seminolipid (SemL) and phosphatidylcholine (PC). No significant differences were found between spermatozoa with high membrane fluidity or low membrane fluidity (*n* = 6 stallions). **(B)** Similarly, the comparison of cholesterol and desmosterol levels in low and high membrane fluidity populations revealed no significant difference in major sterol content in stallion spermatozoa (*n* = 6 stallions). Abbreviations: SM, sphingomyelins; SemL, seminolipids; PS, phosphatidylserines; PI, phosphatidylinositols; PE, phosphatidylethanolamines; PC, phosphatidylcholines; PA, phosphatidic acids; lysoPS, lysophosphatidylserines; lysoPI, lysophosphatidylinositols; lysoPG, lysophosphatidylglycerols; lysoPE, lysophosphatidylethanolamines; lysoPC, lysophosphatidylcholines; Hex2Cer, dihexosylceramides; HexCer, hexocylceramides; Cer, ceramides.

Given that the cryo-ET analysis revealed modulated membrane organization in sperm cells with high membrane fluidity, we next sought to determine whether changes in membrane organization were reflective of changes in either the phospholipidome or alterations in cholesterol efflux caused by bicarbonate treatment. While lipidomic analysis of FACS-sorted spermatozoa revealed 15 distinct phospholipid and glycolipid classes (Figure 5A) consisting of >150 unique phospholipid/glycolipid species (Supplementary File S1), no significant differences in the relative intensity of these lipid classes, or in individual molecular species, were detected between low and high membrane fluidity stallion sperm samples (*n* = 6 stallions; Figure 5A). Similarly, when the levels of cholesterol and desmosterol were measured in the same samples, equivalent pmole amounts/sample (∼500,000 sperm cells) were detected in low and high membrane fluidity sorted cell populations (Figure 5B). The absence of differences in lipid composition between the high and low membrane fluidity sperm subpopulations indicates that differences in membrane fluidity are not caused by compositional differences in sperm membrane lipids. Therefore, further experiments were carried out to investigate whether or not the organization of lipids in the sperm membrane were altered in a cAMP-dependent manner.

### Flow Cytometry Detects PS, but Not PE Exposure in Viable Stallion Sperm After Bicarbonate Stimulation

**FIGURE 6:**
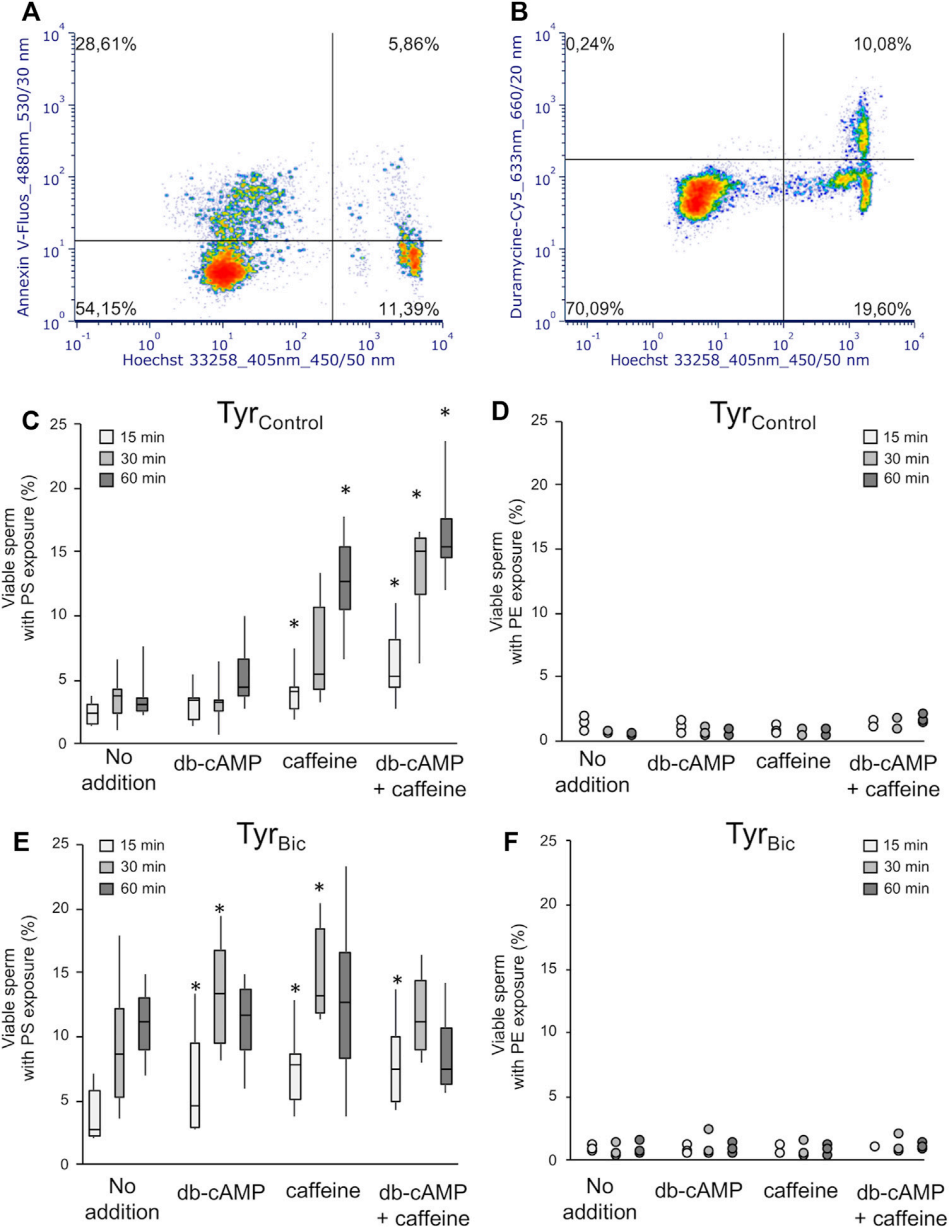
Viable stallion spermatozoa demonstrate exposed PS, but not PE upon direct or indirect elevation of intracellular cAMP levels. Spermatozoa were incubated either in the presence of 30 mM bicarbonate (Tyr_Bic_) or its absence (Tyr_Control_). Media contained either no further additions, 1 mM db-cAMP, 1 mM caffeine, or a combination of 1 mM each of db-cAMP and caffeine. PS exposure to the outer lipid monolayer of the sperm plasma membrane in viable (Hoechst 33258 negative) sperm was detected using Annexin V-Fluos [**(A,C,E)**; *n* = 6 stallions]. An asterisk (*) indicates significant differences between samples with no addition and samples exposed to db-cAMP and/or caffeine (*p* < 0.05). PE exposure residues was monitored using duramycine-Cy5 [**(B,D,F)**; *n* = 3 stallions].

In the sperm cells of most species studied, rearrangement of the sperm lipid membrane bilayer is essential to increase its fluidity prior to fertilization (Visconti et al., 1995a; Gadella and Harrison, 2000; Cross, 2003). The following experiment was performed to understand whether the exposure of PS and PE phospholipids is required for the activation of the sAC/cAMP/PKA pathway leading to an increase in membrane fluidity as previously described in boar sperm (Flesch and Gadella, 2000). In a preliminary experiment we observed that M540 staining may have a small, but significant impact on the number of viable spermatozoa detected as Annexin-V positive (data not shown). Consequently, the following results for PS and PE exposure in viable spermatozoa were obtained in the absence of M540 staining so as not to bias the analysis (Figure 6). Samples with M540 staining were run in parallel to ensure that most of the viable spermatozoa were showing increased membrane fluidity. Annexin-V labelling demonstrated that PS exposure could be detected in Tyr_Bic_ in a subset of viable spermatozoa (Figure 6A). However, exposure of PE in viable spermatozoa was barely observed (Figure 6B).

Bicarbonate was required to significantly increase the proportion of viable sperm with PS exposure after 30 and 60 min incubation (Tyr_Control_ (no addition) Figure 6C versus Tyr_Bic_ (no addition) Figure 6E). Maximal PS exposure in Tyr_Bic_ (no addition) was reached after 60 min incubation (Figure 6E). Caffeine, db-cAMP, and the combination of both compounds were able to increase the population of viable spermatozoa with PS exposure after 15 min and/or 30 min in Tyr_Bic_, but not after 60 min (Figure 6E). In the absence of bicarbonate (Tyr_Control_), either caffeine or the combination of caffeine and db-cAMP initiated an increase in the percentage of viable sperm with PS exposure (Figure 6C). Values in Tyr_Control_ with db-cAMP and caffeine were identical to those from Tyr_Bic_ (no addition; Figures 6C,E). The exposure of PE in viable sperm was always limited to less than 5% of the spermatozoa and could not be stimulated by db-cAMP, caffeine, or the combination of the two compounds (Figures 6D,F).

### Live Imaging Revealed Distinctly Different Staining Patterns for Annexin V in Viable Stallion Sperm

**FIGURE 7:**
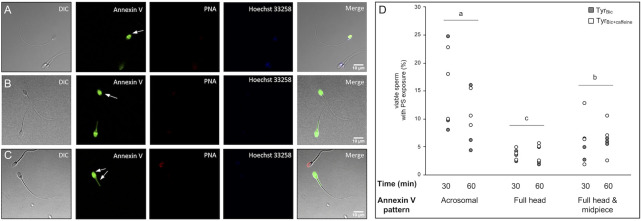
Patterns of phosphatidylserine-exposure in viable stallion sperm. Spermatozoa were incubated either in the presence of 30 mM bicarbonate (Tyr_Bic_) or Tyr_Bic_ containing 1 mM caffeine (Tyr_Bic+caffeine_). An exposure of phosphatidylserine (PS) in viable (Hoechst 33258 negative) sperm was detected using Annexin V-Fluos (*n* = 3 stallions). PNA-Alexa Fluor647 was included as an additional marker for acrosome integrity. Staining patterns for Annexin V were observed in the acrosomal area [**(A)**, arrow], across the complete head region [**(B)**, arrow] or in the complete head and the midpiece [**(C)**, arrows]. The frequency of each pattern in the viable sperm population was determined after 30 and 60 min incubation time **(D)**. Differing letters **(A–C)** indicate significant differences between the abundance of each labelling pattern for Annexin V. There were no significant differences between timepoints (*n* = 12 samples per pattern; *p* < 0.05).

As flow cytometry cannot specify where Annexin-V binds within cells, live imaging was used to visualize Annexin-V staining patterns in stallion sperm under capacitating conditions in Tyr_Bic_. Three main Annexin-V staining patterns were observed in viable stallion spermatozoa (Figure 7; arrow indicated). The dominant pattern was an homogenous labelling of the acrosomal area in the sperm head (Figure 7A), followed by a labelling of the entire head and midpiece (Figure 7C). A smaller subset of spermatozoa stained Annexin-V positive over the whole sperm head (Figures 7B,D). Statistical analysis revealed significant differences in the abundance of these three staining patterns (i.e., number of cells for each Annexin-V labelling pattern), but not significant influence of time or treatment on the number of cells demonstrating each pattern.

## Discussion

Failure of *in vitro* capacitation of stallion sperm has been cited as a major limiting factor in the development of conventional IVF in the horse (Tremoleda et al., 2003). This means that equine IVF in practice is currently achieved *via* intracytoplasmic sperm injection (ICSI), a time-consuming process that requires highly-trained individuals and expensive equipment (Stout and Griffiths, 2021). Although previous studies have reported successful induction of sperm-zona pellucida interaction between equine gametes (Macías-García et al., 2015), media developed to support gamete interaction have not yielded repeatable IVF success (Choi et al., 1994; Dell’Aquila et al., 1997a; Dell’Aquila et al., 1997b; Alm et al., 2001; Hinrichs et al., 2002; Mugnier et al., 2009). Unlike other mammalian species such as the mouse and the boar, stallion sperm membrane physiology and lipid biochemistry have not been meticulously explored to understand essential species-specific events required for capacitation.

Rather than seeking to develop a new capacitation medium, our study aimed to improve understanding of well-described capacitation events, such as bicarbonate-driven membrane destabilization, through the use of advanced technologies including cryo-electron tomography, phospholipidomics, and live imaging. Many of these techniques have not previously been applied to study capacitation in stallion spermatozoa. Assessing stallion sperm capacitation in more intricate detail yielded new insight into the timing of and requirements for capacitation. Specifically, we now know that the membrane remodelling induced by bicarbonate promotes rapid fluidization of the membrane (within 15 min of bicarbonate exposure) that is driven, at least in part, by sAC. Moreover, this extensive bicarbonate-induced membrane reorganization that can be visualized by cryo-electron tomography does not require overt changes in the overall phospholipid composition of the membrane, nor does it involve detectable sterol efflux. These results are in stark contrast to the membrane fluidization process in boar spermatozoa where a redox-dependent sterol efflux facilitates downstream membrane changes (Boerke et al., 2013). We will further discuss the peculiarities of the capacitation of stallion spermatozoa and how future research could be designed to further understand this process and aid development of equine assisted reproduction.

To become capable of fertilizing an oocyte, sperm cells must pass through capacitation steps that permit the plasma membrane to transition to a metastable, fusible state (Gadella and Harrison, 2002; Maitan et al., 2021). *In vitro*, this process can be achieved using capacitating media in which bicarbonate is a key element. An early alteration induced by bicarbonate in sperm membranes is an increase in membrane phospholipid packing disorder that can be detected by the fluorescent amphiphilic probe M540 (Harrison, 1996). Indeed, the increase in the live, M540 positive sperm population induced by Tyr_Bic_ media in our study indicates that this capacitation step can be rapidly achieved in a large proportion of stallion sperm by exposure to bicarbonate. Moreover, when intracellular cAMP levels were increased by the inclusion of db-cAMP and caffeine, this M540 response could be further amplified. This confirms earlier reports that this step in capacitation is important for stallion sperm, as it is for sperm from other mammalian species (pig: Harrison, 1996; stallion: Rathi et al., 2001; dog: Steckler et al., 2015). By inhibiting sAC using LRE1, we demonstrated the involvement of sAC in the regulation of cyclicAMP that underpins the bicarbonate-membrane fluidity response; the incubation of stallion sperm with LRE1 resulted in a dose-dependent decrease in membrane fluidity. In contrast to boar spermatozoa, sAC was localized across the acrosomal region of stallion spermatozoa suggesting that the precise role of sAC in stallion sperm may differ to boar sperm (Leemans et al., 2019b). sAC has previously been linked to the capacitation-related rearrangements of lipids in human sperm (de Vries et al., 2003), as well as to tyrosine phosphorylation (Wertheimer et al., 2013).

Having confirmed that bicarbonate can induce a significant, sAC-regulated, M540 response in stallion spermatozoa, we examined the nature of the increased membrane fluidity using a combination of fluorescence-assisted cell sorting and cryo-electron tomography. This approach allowed us to distinguish between viable “low membrane fluidity” cells and viable “high membrane fluidity” cells and to determine how they differ morphologically. Stallion spermatozoa from the “high membrane fluidity” population consistently showed evidence of membrane destabilization, including vesiculation, rupture, and/or swelling. In contrast, the “low membrane fluidity” cells mostly had intact membranes. Importantly, these observations were not an artifact of the sorting process. Although the sorted “low fluidity” population had more cells with disrupted membranes than the non-sorted control, the majority of sperm had intact membranes, as opposed to the sorted “high fluidity” population in which most cells showed signs of membrane destabilization. Furthermore, sorted “low fluidity” and non-sorted control cells were very similar in terms of membrane morphology, with smooth and regularly-spaced PM and OAM. These observations indicate that extensive membrane remodelling takes place in response to bicarbonate, and is an important step on the path to the acrosome reaction.

Given the membrane destabilization observed in the tomograms, we anticipated that this lipid remodelling was likely to be accompanied by changes in phospholipid and/or sterol content in the sperm membranes. However, investigation of the phospholipidome of FACS sorted “high membrane fluidity” and “low membrane fluidity” spermatozoa revealed no significant differences in the abundance of phospholipid classes or sub-species. Moreover, no significant difference in the content of cholesterol or desmosterol, the key sterols in sperm membranes, were detected between the two defined cell populations. These observations do not align with our previous understanding of sperm membrane remodelling in which the addition of albumin to capacitating media supported cholesterol efflux from bicarbonate responsive cells (Flesch and Gadella, 2000). In some species, depletion of cholesterol from the plasma membrane occurs after the formation of oxysterols, which activate sterol transporter proteins (such as albumin; Brouwers et al., 2011; Boerke et al., 2013). While the measurement of oxysterols in stallion sperm remains to be performed, the retention of membrane cholesterol in stallion sperm cells implies that bicarbonate-induced membrane remodelling is likely to occur through novel mechanisms in this species. Importantly, an inability to withdraw membrane cholesterol from stallion spermatozoa has also been noted in independent studies (Macías-García et al., 2015). An alternate explanation may be that bicarbonate-enriched media such as Tyr_Bic_ are insufficient to trigger observable cholesterol removal from stallion sperm membranes. While the latter remains to be explored, Tyr_Bic_ is known to be sufficient to induce several downstream events of stallion sperm capacitation, such as responsiveness to acrosome reaction inducing stimuli. The acrosome reaction is however thought to be permitted by cholesterol efflux (Cross, 1998; Boerke et al., 2008). It is possible that in stallion spermatozoa, membrane remodelling can occur despite the retention of sterols. However, these aspects should be explored further in detailed lipidomic studies focused on understanding cholesterol acceptors that may be unique to stallion spermatozoa. Nonetheless, this observation marks a distinct aspect of stallion sperm capacitation that should be considered when designing future IVF media for equine gametes.

In some species, the increase in membrane phospholipid disorder that is detected by M540 leads to an activation of a phospholipid scramblase which in turn collapses the lipid asymmetry across the plasma membrane. As a result, PE and PS are translocated to the outer surface of spermatozoa and can be detected using fluorescent probes (Williamson and Schlegel, 1994; Gadella and Harrison, 2002). Incubating boar sperm for 2 h in a bicarbonate containing medium has previously revealed exposure of PS in a substantial subpopulation of intact cells, reaching a maximum at 60 min of incubation (Gadella and Harrison, 2002). Similarly, incubating human sperm with bicarbonate for 4 h resulted in steady state labelling of PS with Annexin V after 90 min of incubation (de Vries et al., 2003). However, some contradictory evidence gathered for boar spermatozoa has also suggested that PS externalization may identify the non-viable sperm population (Kurz et al., 2005). In our experiments, 60 min of incubation in Tyr_Bic_ medium, induced exposure of PS in only 12% of stallion spermatozoa. However, inclusion of a viability stain confirmed that this population consisted of live cells. In stallion spermatozoa, the PS exposure was found to take place at the external surface of the plasma membrane after 30 min of incubation with bicarbonate and thus, temporally, may follow the collapse (or scrambling) of the plasma membrane phospholipid asymmetry that appears to occur following only 15 min of incubation in Tyr_Bic_. Changes in the transbilayer movement of phospholipids are also controlled through the cAMP-dependent phosphorylation pathway that results in the increase in M540 positive cells (Gadella and Harrison, 2000; Harrison and Miller, 2000). Caffeine, whether in combination with db-cAMP or not, also induced an increase in the percentage of live, M540 and Annexin-V positive cells.

With respect to the Annexin-V staining, fluorescence in the acrosome region only was more prevalent than other staining patterns in media containing bicarbonate. Gadella and Harrison (2002) previously demonstrated in boar sperm cells that Annexin V labelling was restricted to the anterior acrosomal region of the sperm head. Moreover, they demonstrated that Annexin-V labelling of the midpiece of the sperm cell was indicative of propidium iodide positive cells (dead or dying). The three staining patterns we observed for Annexin-V in stallion sperm suggest a sequential evolution of PS exposure, with the first step in membrane remodelling resulting in externalization of PS in the acrosomal region. Notably, as some Annexin-V stained cells were also labelled positively for PNA, these cells may have possessed compromised acrosome integrity, potentially allowing annexin V to interact with the inner acrosomal membrane. Thus, this could represent the second step in the evolution of PS exposure. However, the acrosomal status of these cells needs to be further examined in a quantitative manner. In accordance with the previous literature in boar sperm, the midpiece staining pattern we observed in live stallion sperm may indicate that these cells are in a final stage of PS exposure that will end in cell death if fertilization does not take place soon. This is in accordance with previous proposals that capacitation and cell death are interconnected processes in sperm cells (Aitken, 2011; Aitken and Drevet, 2020).

Contrasting the finding that a very high percentage of viable stallion spermatozoa become M540 positive in the presence of bicarbonate, with the very low percentage of viable sperm that exhibit PS exposure leads us to question whether PS exposure is a critical requirement for capacitation of stallion sperm. This is one of the first manuscripts to report on Annexin-V labelling to assess PS exposure by live imaging rather than confocal microscopy. Thus, a comparative study assessing whether PS exposure is indeed a hallmark of capacitation in other species should be performed using live cell technologies. For stallion spermatozoa, a live imaging time series will assist in understanding the sequence of Annexin-V staining patterns, and any potential link with PNA staining patterns and the acrosome reaction.

**FIGURE 8:**
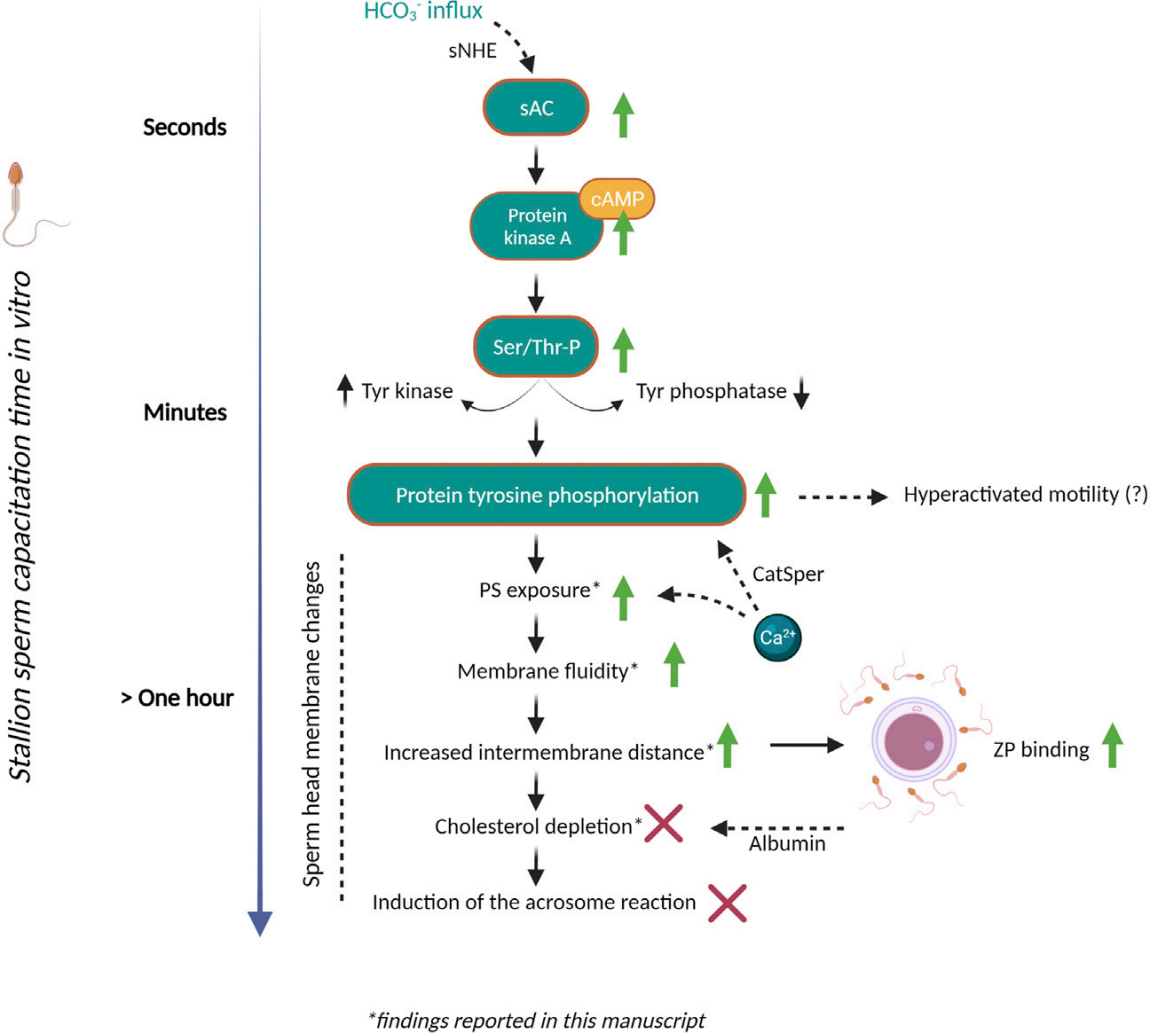
Steps towards stallion sperm capacitation *in vitro* and ongoing challenges. A number of changes must take place in the sperm head membrane to permit interactions with the oocyte. In the stallion we now know that the bicarbonate induced membrane fluidity changes that facilitate downstream sperm functions are regulated by soluble adenylyl cyclase. This leads to an increase in cyclicAMP levels, the regulation of tyrosine kinases and phosphatases and an increase in protein tyrosine phosphorylation (as reported in independent studies). We have demonstrated here that a population of stallion sperm cells also expose phosphatidylserine (PS) in the outer leaflet of the plasma membrane during this process but remain viable. Using cryo-electron tomography we have observed distinct changes in the stallion sperm membrane (such as altered intermembrane distance) in response to bicarbonate. However, these morphological membrane changes were not accompanied by a significant change in membrane phospholipid composition, nor cholesterol efflux. The lack of cholesterol efflux may be a unique aspect of stallion sperm capacitation that differs from other species studied, such as the boar. However, these data may indicate that bicarbonate-enriched capacitation media is insufficient to permit a complete capacitation of stallion sperm cells. Indeed, the field lacks a reliable method to induce acrosomal exocytosis *in vitro*. This incomplete capacitation may underpin our inability to conduct IVF in the horse. Further research should be focused around elucidating the “slower” aspects of the capacitation process that permit the acrosome reaction and sperm-egg fusion. Abbreviations: HCO_3_^−^, bicarbonate; sNHE, sperm-specific Na^+^/H^+^ exchanger; sAC, soluble adenylyl cyclase; cAMP, cyclic adenosine monophosphate; PKA, protein kinase A; Ser/Thr-P, serine/threonine phosphorylation; Tyr, tyrosine. This figure was created with BioRender.com.

In conclusion, a large percentage of stallion spermatozoa demonstrate changes in phospholipid disorder (detected with M540) in bicarbonate containing media, whereas only a relatively small population of viable spermatozoa expose PS. The observation of three different Annexin-V staining patterns for live stallion spermatozoa may warrant further investigation with respect to whether these represent sequential steps in membrane remodelling. However, it is important to note that while this manuscript highlight potential equine-specific features, further work with direct comparative studies is required to accurately delineate species-specific effects from the effects of capacitation media and other factors that dictate the ability of stallion sperm cells to capacitate. Overall, this study reveals several intricacies of the bicarbonate-induced membrane remodelling response in stallion spermatozoa, a key finding being that this remodelling occurs despite retention of membrane sterols and all other lipid components (summarised in Figure 8). Further investigation into sAC inhibition and alternative cholesterol acceptors should help to further delineate key steps in the preparation of equine spermatozoa for fertilization.

## Data Availability

The original contributions presented in the study are included in the article/Supplementary Material, further inquiries can be directed to the corresponding authors. Raw data is publicly available at the following link: https://public.yoda.uu.nl/dgk/UU01/LRWWGJ.html.

## Supplementary Material

Supplementary Figure S1 Stallion sperm membrane reorganization detected by Cryo-EM after incubation with bicarbonate. Spermatozoa were incubated either in the presence of 30 mM bicarbonate (TyrBic) or its absence (TyrControl) for 60 minutes and then FACS-sorted to inspect membrane reorganization using cryo-electron microscopy (cryo-EM). To delineate structures in the cryo-EM frames, the membranes are colour coded to indicate the plasma membrane (PM) (blue), outer acrosomal membrane (OAM) and inner acrosomal membranes (IAM) (orange). Red boxes indicate the sperm cell regions depicted in the frames. Unsorted sperm cells in TyrControl medium demonstrated an intact PM and OAM with a consistent intermembrane distance (A, B). This was also demonstrated in sorted sperm cells in TyrControl (C, D). However, frames from sorted sperm cells incubated in TyrBic reveal rupture of the OAM (E, F) and vesiculation of the PM (E) (n = 3 stallions). (G, H) Boar and stallion sperm cell secondary antibody only controls were prepared where the anti-ADCY10 antibody was replaced by 1% BSA/PBS and cells were then counterstained with Hoechst 33258 and PNA (n = 3 stallions/boars). (I) Percentage of live, M540 positive sperm in different base media with 30 mM bicarbonate (TyrBic). Media were supplemented with either 1 mM db-cAMP, 1 mM caffeine, or both to increase intracellular cAMP levels. Different small letters indicate effects of the supplements in a given medium and at a given time (p < 0.05). All values are presented as mean ± SD.

Supplementary Figure S2 58 negative) sperm was monitored in the absence or presence of KH7 KH7 has an off-target effect on sperm mitochondrial membrane potential during inhibition of sAC. Spermatozoa were incubated either in the presence of 30 mM bicarbonate (TyrBic) or its absence (TyrControl). Media contained either no further additions, DMSO (a solvent control), or increasing concentrations of KH7 to indirectly block sAC activity. An increase in plasma membrane fluidity, i.e., increased merocyanine 540 staining, in viable (Hoechst 332[**(A, B)**; n = 6 stallions]. A hash symbol (#) indicates significant differences between DMSO exposed samples and samples without any addition (P < 0.05). An asterisk (*) indicates significant differences between DMSO exposed samples and samples treated with KH7 (P < 0.05). In a subset of samples, the percentage of viable sperm with high mitochondrial transmembrane potential (MMP) was monitored with the probe JC-1 [**(C, D)**; n = 3 stallions]. Concentrations of 60 μM and 120 μM KH7 significantly reduced the percentage of sperm with high MMP compared to control samples (DMSO; p < 0.05).
