# Supplementary figures and images for: Bicarbonate-Stimulated Membrane Reorganization in Stallion Spermatozoa
Source: Front Cell Dev Biol. 2021 Nov 17;9:772254. doi: 10.3389/fcell.2021.772254 (PMC8635755; doi:10.3389/fcell.2021.772254)

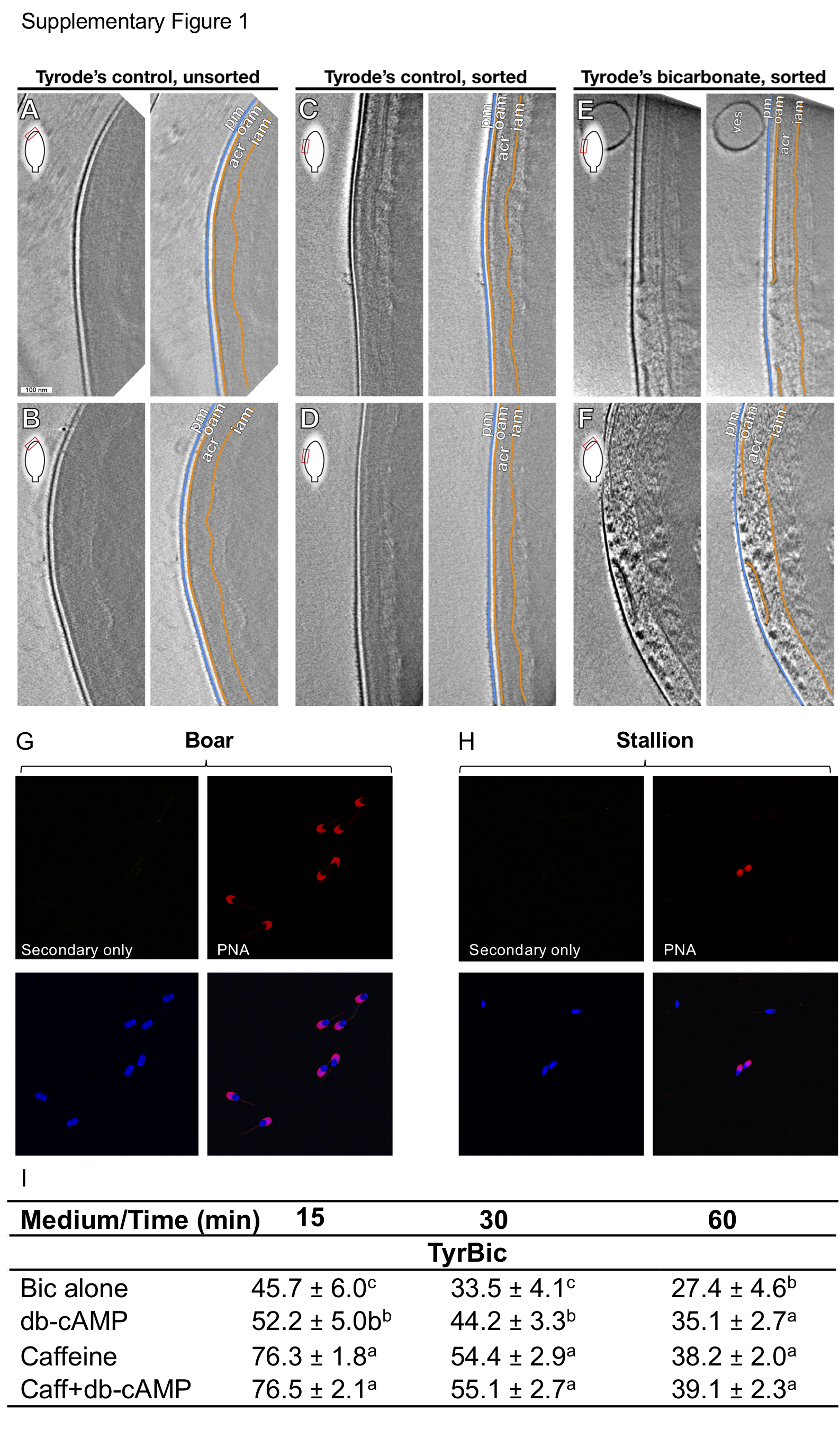

Supplement: Supplementary file 1 [file Image1.tiff]

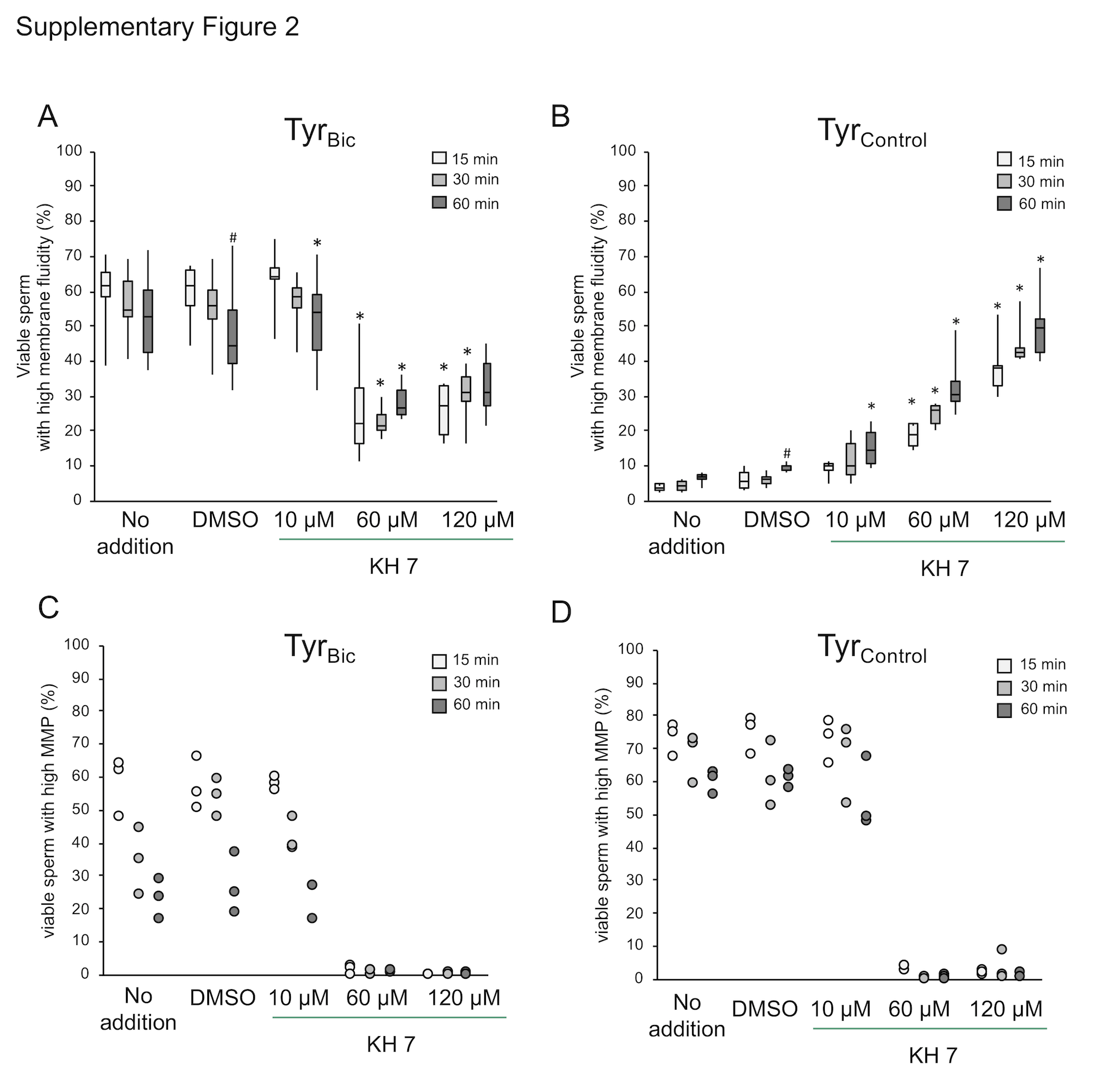

Supplement: Supplementary file 3 [file Image2.tiff]
